# The Role of Proteostasis Derailment in Cardiac Diseases

**DOI:** 10.3390/cells9102317

**Published:** 2020-10-19

**Authors:** Bianca J. J. M. Brundel

**Affiliations:** Department of Physiology, Amsterdam UMC, Vrije Universiteit, Amsterdam Cardiovascular Sciences, 1081 Hz Amsterdam, The Netherlands; b.brundel@amsterdamumc.nl

## Abstract

The incidence and prevalence of cardiac diseases, which are the main cause of death worldwide, are likely to increase because of population ageing and changes in lifestyle. Prevailing theories about the mechanisms of cardiac disease onset feature the gradual derailment of cellular protein homeostasis (proteostasis) and loss of the protein quality control as central factors. In the heart, loss of protein patency, due to flaws in design (genetically) or environmentally-induced wear and tear, may overwhelm protein quality control, thereby triggering derailment of proteostasis and contributing to cardiac disease onset.

This Special Issue describes original research and review papers addressing various aspects of proteostasis derailment, including impairment of chaperones, ubiquitin–proteosomal systems, autophagy, mitophagy and loss of sarcomeric and cytoskeletal proteins to underlie cardiac disease [1].

The contribution of Dorsch is directed at identification of key players within the protein quality control system to underlie hypertrophic cardiomyopathy [2]. They observed, in human heart tissue of patients carrying a mutation in sarcomeric proteins, increased levels of heat shock proteins (HSP) and acetylated α-tubulin compared to mutation negative patients. As acetylated tubulin results in polymerization of microtubules and stabilization of cardiomyocyte structure, the findings suggest a role for HSP in microtubular network proliferation to compensate for sarcomeric loss in mutation-induced hypertrophic cardiomyopathy. In line with these findings, Singh elaborates on the role of the intermediate filament protein desmin in cardiac function [3]. Mutations in desmin result in aggregate formation. These may hamper cardiomyocyte function and lead to cardiac disease onset. On the flipside, desmin aggregates were also found to protect from ischemic and ischemia-reperfusion injury. This discrepancy warrants further investigation on the exact role of aggregates on cardiomyocyte function. Starreveld studied whether the HSP-inducing compound L-glutamine has an effect on HSP levels in patients with atrial fibrillation [4]. Previous studies already revealed that HSP induction protects the heart against atrial fibrillation [5,6]. Indeed, L-glutamine treatment alters serum HSP levels and normalized metabolite levels in patients with atrial fibrillation. The next step will be mounting of a clinical trial to study the effect of L-glutamine on the atrial fibrillation burden.

Emerging evidence indicates a link between cardiac disease and endoplasmic reticulum (ER) stress and mitochondrial dysfunction. A comprehensive overview on protein secretion due to ER stress, and their role on heart function is provided by Meyer [7]. Roles include augmentation of cardiac cell viability, pro- and anti-apoptotic and oncogenic effects. Although, the exact role of these secreted proteins needs to be further explored, for sure, the effects are all of vital importance to heart function. Pires Da Silva investigated the effect of class III histone deacetylases, Sirtuin 1, on autophagy and cardiac function [8]. They found that upon ER stress, Sirtuin 1 prevents cardiac dysfunction by enhancing autophagy.

In addition to ER stress-induced autophagy, ER is in close contact with mitochondria. Li describes the concept of ER and mitochondrial stress and how the interactions between both organelles influence cardiomyocyte function [9]. Loss of contact between ER and mitochondria drives cardiac diseases. In line, Wiersma found that mitochondrial dysfunction underlies atrial cardiomyocyte damage and consequently atrial fibrillation [10]. Furthermore, drugs directed at mitochondrial dysfunction, such as Ru360 and SS31, may have value to treat clinical atrial fibrillation. Interestingly, mitochondrial damage results in the release of mitochondrial DNA into the circulation [11]. Therefore, mitochondrial DNA levels may represent a blood-based biomarker for atrial fibrillation. To expand on the role of mitochondrial function in heart function, Ghosh highlights key findings related to protein and mitochondrial quality control and its decay to underlie cardiac aging [12].

To restore cardiac function, drugs directed at restoration of proteostasis are of interest. Blackwood discusses the role of activating transcription factor 6 (ATF6) in restoration of proteostasis. ATF6 has been shown to induce ER-targeted protein expression and enhances ER protein folding and degradation [13]. Recent findings indicate functions for ATF6 outside of the ER. These functions include enhancement of cardiomyocyte hypertrophy, natriuretic peptide secretion and stimulus dependent unique gene programs. These new roles of ATF6 in cardiac structure and function and novel compounds directed at ATF6 are definitely of interest to further explore in relation to various cardiac diseases.

An important modulator of protein quality control is the ubiquitin-proteasome system, which degrades marked proteins to ensure a healthy cell function. Interestingly, immune cells contain i-proteasome subunits which enhance immune responses to pathogen exposure. Inhibitors of i-proteasomes, such as ONX 0914, are thought to be beneficial in inflammation-driven cardiogenesis and transplant rejection. Neumaier reveals that pretreatment of mice susceptible to acute viral-induced myocarditis with ONX 0914 accelerates infiltration of the virus into heart tissue, thereby promoting chronic cardiac inflammation [14]. In their study, ONX 0914 also inhibits normal proteasome function which is detrimental for cardiomyocyte function. More research is warranted to study the cross-talk between various proteasome systems and drug interactions.

In summary, emerging evidence unravel key roles for molecular pathways to result in proteostasis derailment and as such drive impairment of cardiomyocyte structure and function. This knowledge is important to design novel compounds directed at proteostasis derailment to conserve cardiomyocyte function and combat cardiac disease.
